# Two-Component Systems in *Francisella* Species

**DOI:** 10.3389/fcimb.2019.00198

**Published:** 2019-06-12

**Authors:** Monique L. van Hoek, Ky V. Hoang, John S. Gunn

**Affiliations:** ^1^School of Systems Biology, George Mason University, Manassas, VA, United States; ^2^Center for Microbial Pathogenesis, Nationwide Children's Hospital, Columbus, OH, United States; ^3^Infectious Diseases Institute, The Ohio State University, Columbus, OH, United States; ^4^Department of Pediatrics, The Ohio State University College of Medicine, Columbus, OH, United States

**Keywords:** two-component system (TCS), *Francisella*, response regulator, sensor histidine kinase, QseC, QseB, PmrA, tularemia

## Abstract

Bacteria alter gene expression in response to changes in their environment through various mechanisms that include signal transduction systems. These signal transduction systems use membrane histidine kinase with sensing domains to mediate phosphotransfer to DNA-binding proteins that alter the level of gene expression. Such regulators are called two-component systems (TCSs). TCSs integrate external signals and information from stress pathways, central metabolism and other global regulators, thus playing an important role as part of the overall regulatory network. This review will focus on the knowledge of TCSs in the Gram-negative bacterium, *Francisella tularensis*, a biothreat agent with a wide range of potential hosts and a significant ability to cause disease. While TCSs have been well-studied in several bacterial pathogens, they have not been well-studied in non-model organisms, such as *F. tularensis* and its subspecies, whose canonical TCS content surprisingly ranges from few to none. Additionally, of those TCS genes present, many are orphan components, including KdpDE, QseC, QseB/PmrA, and an unnamed two-component system (FTN_1452/FTN_1453). We discuss recent advances in this field related to the role of TCSs in *Francisella* physiology and pathogenesis and compare the TCS genes present in human virulent versus. environmental species and subspecies of *Francisella*.

## Role of TCS in Bacteria

**Figure 1:**
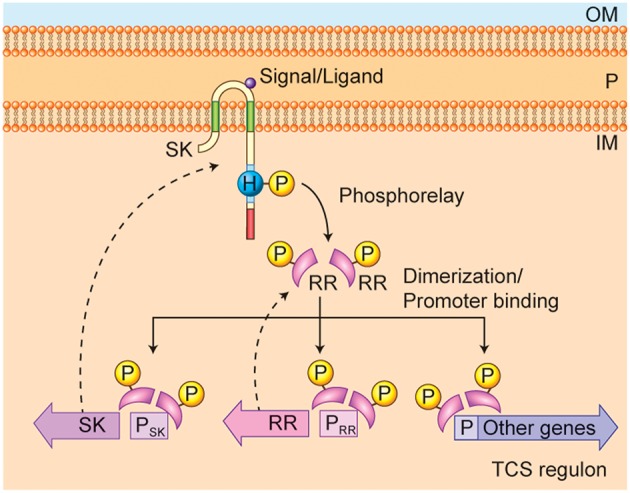
*Francisella* non-classical two-component system (TCS) QseC and QseB/PmrA. The domain organization of the sensor kinase (SK) QseC as a transmembrane protein is shown, along with its critical domains (described in detail in Figure 3) and autophosphorylation phosphoacceptor site on Hisitidine 259. The sensing domain is located in the periplasmic region (P), between the inner membrane (IM) and outer membrane (OM). The signal or ligand is shown in the periplasm as well. The response regulator (RR) QseB/PmrA is shown as pink curved shape, along with the phosphorylated aspartate phosphoacceptor site (P). The phosphorelay from the SK to the RR is shown by the solid curved arrow. Dimerization of the phosphorylated response regulator enables promoter binding. The auto-regulation of the RR QseB/PmrA expression and the regulation of SK QseC expression along with expression of the TCS regulon, is illustrated in the lower part of the figure, with dotted-line arrows to the resulting proteins.

Bacteria alter their gene expression in response to changes in their environment through various mechanisms, including signal transduction systems. These signal-transduction systems use kinases with extracellular or periplasmic sensing domains to transfer phosphate groups to DNA-binding molecules and alter the levels of gene expression. Bacterial signal-transduction systems are typically simpler than the eukaryotic signal transduction systems, often involving only two proteins (a sensing protein and a transcription factor), and thus are called Two-Component Systems (TCS) (Stock et al., 2000; Groisman, 2016). The sensor kinase (SK; a histidine kinase) and the response regulator (RR; a DNA binding protein) make up the two parts of two-component systems (Figure 1).

Two-component systems are known to be involved in bacterial motility and chemotaxis, physiological responses to osmotic changes, biofilm formation, and the regulation of virulence in many bacteria (Stock et al., 2000; Freeman et al., 2013; Groisman, 2016). Two-component systems can also integrate signals from stress pathways, central metabolism and other global regulators, and thus play an important role as part of the overall regulatory network. While both gram-positive and gram-negative bacteria employ TCS to respond to external signals, this review will focus on gram-negative bacteria, and in particular, on *Francisella tularensis*. *Francisella* is a gamma-proteobacteria with a wide range of potential hosts and significant ability to cause disease, yet it has less than 2,000 genes, much less than standard model bacterial organisms, such as *E. coli*. We were interested in surveying the literature on two-component systems in this non-model organism given the many recently sequenced genomes of *Francisella* species, to further our understanding of the bacterial physiology of this organism as well as advances in our understanding of its virulence.

As shown in Figure 1, bacterial sensor histidine kinases (SKs) are typically homo-dimeric transmembrane proteins whose C-terminal tails contain a Histidine phosphotransfer domain and an ATP binding domain. SKs are associated with the bacterial cell membrane and in gram-negative bacteria have a structure that typically consists of three parts: the C-terminal and N-terminal intracellular domains, the periplasmic domain, and the transmembrane domains. The periplasmic domain forms a loop (sensing domain) connecting the two trans-membrane domains in the periplasm which can be long or short. Bacterial TCS SKs are typically histidine kinases, in contrast to eukaryotic signal transduction kinases, which are tyrosine kinases or serine/threonine kinases. The histidine kinase activity is activated by signal or ligand binding, which leads to dimerization and activation of the SK (Stock et al., 2000; Groisman, 2016). The activated histidine kinase autophosphorylates on a critical histidine residue in the intracellular C-terminal domain. This phosphate is then transferred to a critical aspartate residue on the RR by the phospho transferase activity of the SK. When the RR is phosphorylated, it also typically dimerizes, and the active, phosphorylated dimer can then bind to DNA in the promoter region of a regulated gene, and act as a transcription factor to increase or decrease expression of those genes in its regulon (Stock et al., 2000; Groisman, 2016). Some SKs have been found to have phosphatase activity, which is needed to reset the signal transduction pathway.

In classical TCSs, the SKs and the RRs are co-transcribed in an operon, in which the RR is usually the first gene and the SK is the second gene. In non-classical TCSs, the SK and RR may not be co-transcribed in an operon, but may still work together to affect gene expression (Figure 1). Typically, RRs regulate their own expression, and thus the DNA-binding motif (to which the phosphorylated and dimeric RR binds) is often found in the promoter region of its own gene (P_RR_) in addition to the other genes that are regulated by RRs. Furthermore, the SK expression is typically co-regulated by the RR (Groisman, 2016).

In Gram-negative bacteria, two-component systems have been identified that regulate critical functions, such as biofilm formation and virulence (Pruss, 2017). The presence of the outer membrane in gram-negative bacteria and the location of the SK in the inner membrane means that all the ligands for the SK must be able to penetrate into the periplasm. Signal inputs or ligands, such as fatty-acids (e.g., diffusible signal factors [DSF]) or other small molecules are detected within the periplasmic space by the SK sensing domain. This signal is then transmitted across the membrane to activate the intracellular Histidine kinase domain (Figure 1).

In some gram-negative bacteria the two-component systems are more complex, such as the diffusible signal factor (DSF) response system RpfCG in *Xanthomonas campestris* (Ryan et al., 2015; Cai et al., 2017). SK molecules in these systems, such as RpfC can often have complex sensing domains, in this case containing five trans-membrane domains (Ryan et al., 2015). The RpfCG system in *X. campestris* regulates virulence in this important plant pathogen of rice (Cai et al., 2017). While still retaining the overall process of a SK signaling to a DNA-binding molecule, SK domains may be expressed in multiple molecules, such as a histidine transfer domain that is expressed as a separate protein in *Pseudomonas aeruginosa*. In this example, signal transduction from the membrane-associated histidine kinase PA1396 to the DNA-binding regulator PA1397 involves a separate histidine transfer domain protein (Ryan et al., 2015). In model organisms, such as *Escherichia* (*E.) coli*, TCS are well-studied and there are a large number of TCS proteins; as many as 30 histidine kinases and 32 RRs have been identified (Pruss, 2017).

The two-component systems and their activating ligands have not been as well-studied in non-model organisms, such as *F. tularensis*. This review serves to summarize the literature on the TCSs present in *Francisella*, and to explore what is known about their role in *Francisella* physiology and pathogenesis.

## The Genus Francisella

**Figure 2:**
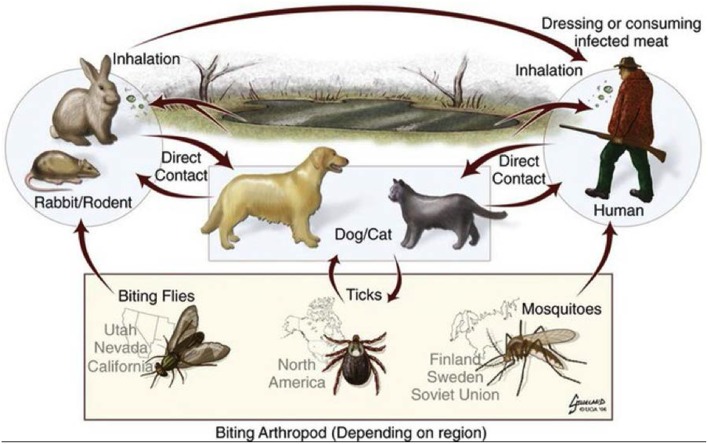
Sylvatic cycle of *Francisella tularensis*, illustrating the transmission cycles and the relevant biting insects depending on the region (Art by Brad Gilleland, UGA College of Veterinary Medicine. © 2004 - 2019 University of Georgia Research Foundation, Inc.). Printed by Permission of the University of Georgia Research Foundation Inc.

Bacteria within the genus *Francisella* are small, gram-negative cocco-bacilli, which are found predominantly in the northern hemisphere (Dennis et al., 2001). These organisms are commonly identified in environmental samples (soil, mud, water), insect vectors (ticks and mosquitoes), single-celled amoeba, fish, birds, and mammals (where it is a facultative intracellular pathogen) (Dennis et al., 2001; El-Etr et al., 2009; Verhoeven et al., 2010). Thus, this organism has three elements to its lifecycle—environmental, vector-borne, and host-associated (Figure 2). Humans are an accidental host for this zoonotic pathogen, but it is highly infectious to humans via aerosol, with <100 organisms as the infectious dose via inhalation (Dennis et al., 2001). The main species of *Francisella* include *F. tularensis*, which contains the human pathogenic strains, *F. novicida* and *F. philomiragia* (Zeytun et al., 2012), which are environmental strains and not typically human pathogens, and *F. noatunensis*, which is a pathogen of fish (Zerihun et al., 2011).

Tularemia (or “rabbit-fever”) is caused by *F. tularensis*. *Francisella* can be transmitted by biting insect vectors, direct contact or consumption of infected animal tissue or contaminated water, or inhalation of aerosolized bacteria (Figure 2) (Dennis et al., 2001). When acquired by humans, tularemia patients demonstrate symptoms of high fever, myalgia, malaise, and primary or secondary pneumonia. Due to its high infectivity to humans via inhalation and its historical development as a biological weapon, *F. tularensis* is categorized as a Category A/Tier 1 biothreat agent (Dennis et al., 2001). Upon necrotic examination of infected animals, such as rabbits or squirrels caught by hunters, miliary spots on the liver along with enlarged and infected reticuloendothelial organs (such as the spleen) are often observed. Upon more detailed examination of infected animals, a large number of bacteria are found within the cells of these organs—including hepatocytes and immune cells, such as macrophages and dendritic cells (Rasmussen et al., 2006)—indicating the role of intracellular replication in the disease course. Bacterial genes that are required for intracellular replication of this organism are considered virulence factors, and are usually associated with virulence in animal infection models as well. Some bacterial genes may therefore be associated with host-infection and virulence, while other genes may be associated with the vector-borne or environmental phases of the *Francisella* life-cycle.

## TCS Genes Present in the Genus *Francisella*

While there have been 30 or more paired two-component systems identified in *E. coli* and other well-studied model organisms and pathogens (Yoshida et al., 2015), very few TCS genes have been identified in the non-model organism *Francisella*. The two-component systems that are currently known in the genus *Francisella* are summarized in Table 1. This table names the TCS genes identified in the most commonly studied species within the genus *Francisella*, including a human virulent strain (*F. tularensis* SchuS4), the live vaccine strain (*F. tularensis* LVS), and two environmental species, *F. novicida* and *F. philomiragia* (Larsson et al., 2005). A more extensive listing of TCS genes identified in the genus *Francisella* is presented in Supplemental Table 1.

**Table 1:** Representative TCS genes in *Francisella* species.

	**Response regulator**			**Sensor kinase**		
	**KdpE**	**Unnamed RR**	**QseB**	**KdpD**	**Unnamed SK**	**QseC**
*F. tularensis* SchuS4	Pseudogene (FTT1735c)	FTT1543	FTT1557c	FTT1736c	Pseudogene (FTT1544)	FTT0094c
*F. tularensis* LVS^*^	Pseudogene (FTL1876)	Pseudogene	FTL0552	Pseudogene	Pseudogene	FTL1762
*F. novicida* U112	FTN1714	FTN1452	FTN1465	FTN1715	FTN1453	FTN1617
*F. philomiragia* 25017	Fphi0890	Fphi1222	Fphi1209	Fphi0888	Fphi1221	Fphi1001

* *FSC200 has similar genes and pseudogenes to LVS, not shown. Gene locus numbers are indicated (Larsson et al., 2005; Rohmer et al., 2007). Pseudogenes are highlighted in gray and the pseudogene locus numbers are in parentheses*.

From Table 1 it can be seen that there are both paired and orphan TCSs in *Francisella* species. The genomes of all strains of *Francisella* examined so far contain the orphan RR QseB (also called PmrA) and the SK QseC, discussed in detail below. The putative potassium sensing TCS, KdpDE, is also present and complete in the environmental strains *F. novicida and F. philomiragia*. The RR KdpE is a pseudogene in *F. tularensis* Schu S4 and *F. tularensis* LVS. A pseudogene is a gene that is related to a functional gene but due to the accumulation of premature stop-codons or frame-shifts is not likely to result in a functional protein product (Champion et al., 2009). The *holarctica* strains of *F. tularensis*, including the live vaccine strain (LVS) and human pathogenic strain FSC200, are also missing the cognate SK, KdpD (Figure 4). There is an unnamed and uncharacterized RR present in *F. tularensis* SchuS4 (FTT_1543), *F. novicida* (FTN_1452), and *F. philomiragia* (Fphi1222), which possesses its cognate, paired SK only in *F. novicida* (FTN_1453) and *F. philomiragia* (Fphi1221). The *Francisella* genome has significant rearrangements and other genomic alterations between the strains, and a large number of pseudogenes are found in the human pathogenic strains, *F. tularensis* SchuS4, and *F. tularensis subsp. holarctica* (Dempsey et al., 2006; Petrosino et al., 2006) (Table 1).

## Response Regulator QseB/PmrA

**Figure 3:**
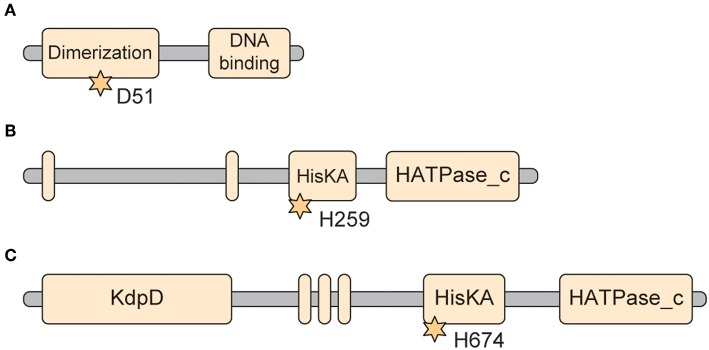
Predicted protein domains of selected Francisella TCS genes. **(A)** QseB. Domain arrangement of *Francisella* QseB/PmrA (FTT_1557c). This 228 amino acid protein is a response regulator. The critical aspartate that receives the phosphorylation is D51 (*). The dimerization domain is from residues 3–113. The DNA binding domain is from residues 147–221 (https://www.uniprot.org/uniprot/Q5NER1; http://pfam.xfam.org/protein/Q5NER1). **(B)** QseC. Domain arrangement of *Francisella* QseC (FTT_0094c). This 475 amino acid protein is a sensor Histidine kinase. This protein is predicted to have two transmembrane domains (Residues 12–36 and 172–195), flanking the sensing domain (33–171). The Histidine kinase domain is located between residues 249–470. The HisKA domain is identified as between 249–314 (also identified as the dimerization domain), while the HATPase_c domain is identified between residues 363–470. The critical phosphoacceptor Histidine involved in phosphorelay is His259 (*) (https://www.uniprot.org/uniprot/Q5NIH6; http://pfam.xfam.org/protein/Q5NIH6). **(C)** KdpD. Domain arrangement for *Francisella* KdpD (FTT_1736c). This 893 (100.9 kDa) protein is a more complex sensor Histidine kinase than QseC. Unlike QseC, it has a long N-terminal domain that is annotated to contain a KdpD domain (from residues 21–230). KdpD also is annotated to have three transmembrane domains (residues 405–435, 442–461, and 467–495), comprising DUF4118. The C-terminal hisitidine kinase domain is annotated from residues 671–890, with the HisKA domain from residues 664–732, and the HATPase_c domain from residues 776–890. The critical Histidine for phosphorelay is at Histidine 674 (*) (https://www.uniprot.org/uniprot/Q5NEA7, http://pfam.xfam.org/protein/Q5NEA7).

The RR that is present in all members of the genus *Francisella* is QseB/PmrA (FTN_1465/FTT1557c) (https://www.uniprot.org/uniprot/A0Q7W8). This 228 amino acid (25.5 kDa) protein contains the critical aspartate residue (D51) that when modified to 4-aspartylphosphate is important in dimerization and DNA binding (Figure 3A). The dimerization domain is annotated from residues 3–113. The DNA binding domain is annotated to be from residues 147–221 (http://pfam.xfam.org/protein/Q5NER1, https://www.ebi.ac.uk/interpro/protein/A0Q7W8). The crystal structure of the RR receiver domain of this protein has been solved to 2.49 A (PDB 5UIC) (Milton et al., 2017) revealing new insights into this molecule and identifying potential therapeutic interventions that target its interactions.

## Sensor Kinase QseC

The SK that is present in all organisms in the genus *Francisella* is QseC (FTT_0094c), part of a putative quorum sensing two-component system. The 475 amino acid protein (54.8 kDa) contains two transmembrane domains flanking the periplasmic sensor domain, and a long cytoplasmic tail containing the Histidine kinase domain and the site of phosphorylation H259 (Figure 3B). The two transmembrane domains are annotated from residues 12–36 and from 172–195, leaving a 136 aa periplasmic loop for the “sensing domain.” The “HisKA” phosphoacceptor domain is from residues 249–314 (also annotated as the dimerization domain), while the “HisATPase_c” Kinase domain is in the c-terminal tail, from residues 363–470 (https://www.uniprot.org/uniprot/Q5NIH6); (https://www.ebi.ac.uk/interpro/protein/Q5NIH6).

## QseB/PmrA and QseC in Virulence and Bacterial Phyiology

TCSs regulate the expression of genes in pathogenic bacteria involved in diverse phenotypes including stress response, antibiotic resistance, cell growth, biofilm formation, and virulence (Stock et al., 2000). In many bacteria deletion of genes encoding various TCS genes results in attenuation in animal models (Eguchi and Utsumi, 2008; Mitrophanov and Groisman, 2008). The role of QseB/PmrA in *Francisella* virulence has been well-established. It has been shown that a QseB/PmrA mutant is avirulent in the macrophage infection model as well as in the murine infection model for *F. novicida* (Mohapatra et al., 2007). Several studies demonstrated that inactivation of the unpaired QseC and QseB/PmrA genes dramatically reduces *Francisella* virulence in macrophages and in a mouse model of tularemia (Mohapatra et al., 2007; Bell et al., 2010) (Table 2). It is not yet known if these two genes act as a functional paired TCS, but as the data described above suggests, the orphan SK KdpD can also participate in a phosphorylation cascade with QseB/PmrA (Bell et al., 2010). In *F. tularensis* SchuS4, expression of QseB (FTT_1557c) is highly upregulated in serine hydroxamate treated cells, a condition that induces active stringent response (Murch et al., 2017). QseB/PmrA has also been shown to be required for biofilm formation in *F. novicida* (Durham-Colleran et al., 2010).

**Table 2:** *Francisella* two-component system genes with a role in virulence.

**Gene (Type/Name)**	**Role in intra-macrophage growth**	**Role in murine infection**	**Role in other systems**
***Francisella tularensis SchuS4***			
FTT_0094c (QseC)		Weiss et al., 2007	Fischer 344 Rat (Ireland et al., 2019)
FTT_1543 (Unnamed RR)			
FTT_1557c (PmrA/QseB)			Upregulated under stress (Murch et al., 2017)
FTT_1736c (KdpD)		Weiss et al., 2007	
***Francisella holarctica*** **LVS**			
FTL_0552 (PmrA/QseB)	Sammons-Jackson et al., 2008	Sammons-Jackson et al., 2008	
FTL_1762 (QseC)			
***Francisella novicida***			
FTN_1452 (Unnamed RR)			Drosophila (Moule et al., 2010)
FTN_1453 (Unnamed SK)			
FTN_1465 (PmrA/QseB)	Mohapatra et al., 2007; Bell et al., 2010	Mohapatra et al., 2007; Bell et al., 2010	Drosophila (Moule et al., 2010)
FTN_1617 (QseC)	Dean and van Hoek, 2015		Waxworm (Dean and van Hoek, 2015)
FTN_1714 (KdpE)			
FTN_1715 (KdpD)	Richards et al., 2008		Drosphila (Moule et al., 2010); Polymyxin resistance (Sampson et al., 2014)

The mechanism by which QseB/PmrA exerts its effect on the regulation of expression of the *Francisella* Pathogenicity Island (FPI) proteins—and thus regulation of virulence—has been studied. QseB/PmrA phosphorylation promotes its association with MglA/SspA, and thus affects the expression of the virulence genes on the FPI (Bell et al., 2010). Interestingly, QseB/PmrA can be phosphorylated by the SK KdpD in this system (Bell et al., 2010), suggesting that there is some promiscuity possible between the SKs and the target RRs. This may promote the survival of *Francisella* strains that lack different members of the TCS.

The SK QseC is required for virulence in *F. tularensis* SchuS4 (Weiss et al., 2007; Rasko et al., 2008; Ireland et al., 2019). *Francisella* QseC was shown to functionally complement a deletion of QseC in *E. coli* (Rasko et al., 2008). In addition, the use of an inhibitor of QseC, LED209, was able to significantly protect mice from a lethal infection by *F. tularensis* SchuS4 (Rasko et al., 2008), and LED209 significantly reduced the expression of several genes in the FPI, including *iglC*. These results suggest that LED209-reduced FPI expression may be associated with the reduced virulence (Rasko et al., 2008). The molecular target of LED209 in the *E. coli* QseC has been identified to be the allosteric modification of two lysines, and in *Francisella* the analogous residue is K269 in QseC (Curtis et al., 2014), placing it in the dimerization- phosphor-acceptor domain. Mutation of the analogous lysine K256 to an arginine inhibited its ability to activate expression of Enterohemorrhagic *E. coli* virulence genes in *E. coli* QseC (Curtis et al., 2014). While these two genes are only about 57% similar, the data in the *E. coli* model and the functional substitution of *Francisella* QseC in *E. coli* suggests that they may have a similar response to and inhibition by LED209.

As mentioned above, inactivation of QseC dramatically reduces *Francisella* virulence in macrophages and in a mouse model of tularemia (Rasko et al., 2008; Mokrievich et al., 2010). In addition, a recent study in Fischer 344 rats demonstrated that insertional inactivation of QseC led to a significant reduction in the numbers of *Francisella tualrensis* Schu S4 in the rat spleen (Ireland et al., 2019). Transposon-insertion mutants of QseC in *F. novicida* were found to be defective for the production of IglC (a critical virulence factor protein in the FPI) and were avirulent in the waxworm infection model (Dean and van Hoek, 2015), as well as being defective in biofilm formation (Durham-Colleran et al., 2010; Dean and van Hoek, 2015). A QseC mutant in *Francisella* vaccine strain 15 (similar to LVS) is reported to have growth defects on solid media, loss of ability to replicate in macrophages, and loss of virulence (Mokrievich et al., 2010). It has also been reported that these QseC mutants have altered LPS structures due to a lack of the high-molecular-mass O-polysaccharide (Mokrievich et al., 2010). Overall, there is significant data to suggest an important role for the sensor kinase QseC in *Francisella*.

## Potassium Regulatory TCS KdpDE

In many gram-negative bacteria, the TCS KdpDE plays an important role in regulating potassium (K+) transport as well as maintaining K+ homeostasis through its regulation of the *kdpFABC* operon and thus expression of the potassium transporter (Freeman et al., 2013). It has been proposed that KdpDE TCS is also responsive to bacterial stress, quorum sensing and other stressors of pathogens, such as phagocytosis (Freeman et al., 2013). In addition, the RR KdpE may be a direct regulator of virulence factor expression through its DNA binding activity, suggesting additional potential roles for KdpDE in virulence and pathogenesis (Freeman et al., 2013). In *E. coli*, the activating stimulus of KdpD is thought to cause an inactivation of its phosphatase activity, leading to increased phosphorylation of KdpE and increased transcription and expression of the high-affinity K+ transporter (Freeman et al., 2013). Also, as mentioned previously, KdpD was able to phosphorylate QseB/PmrA, and regulate FPI gene expression in association with a global regulator MglA/SspA (Bell et al., 2010).

KpdD in *F. tularensis* SchuS4 (FTT_1736c) is a more complex Sensor Kinase than QseC. In particular, it has a longer C-terminal domain compared to QseC, containing the “KdpD” domain. It is a large 893 residue bacterial integral membrane protein of 100.9 kDa, predicted to have three transmembrane domains and a Histidine kinase domain in the c-terminal tail (Residues 671–890) (Figure 3C) (https://www.uniprot.org/uniprot/Q5NEA7). There is an annotated KdpD domain from residues 21–230. The predicted phosphorylation site on KdpD is Histidine 674 (https://www.ebi.ac.uk/interpro/protein/Q5NEA7, http://pfam.xfam.org/protein/Q5NEA7,https://www.uniprot.org/uniprot/Q5NEA7).

The SK KdpD is present in the environmental strains of *Francisella*, as well as in *F. tularensis* SchuS4, the highly virulent strain (Tables 1, 3). *Francisella* KpdD is often annotated as an “osmosensitive K+ channel Histidine kinase” or “Universal stress family protein” (Uniprot.com). Interestingly, the environmental strains of *Francisella*, such as *F. philomiragia* and *F. noatunensis* are found in brackish water and seawater, respectively (Mikalsen et al., 2009; Propst et al., 2016), which may exert osmotic stress on those organisms.

**Table 3:** Kdp operon in *Francisella novicida* U112 compared to *F. tularensis SchuS4*.

**Locus**	**Gene**	**Description**	**Presence in *F. tularensis SchuS4***
FTN_1714	*kdpE*	Response regulator transcription factor	Pseudogene in SchuS4 (https://www.ncbi.nlm.nih.gov/gene/3192093)
FTN_1715	*kdpD*	Sensor Histidine kinase	Present (https://www.ncbi.nlm.nih.gov/gene/3192246)
FTN_1716	*kdpC*	Potassium-transporting ATPase C chain	Present (https://www.ncbi.nlm.nih.gov/gene/3192156)
FTN_1717	*kdpB*	Potassium-transporting ATPase B chain	Present (https://www.ncbi.nlm.nih.gov/gene/3192083)
FTN_1718	*kdpA*	Potassium-transporting ATPase subunit A	Pseudogene in SchuS4 (https://www.ncbi.nlm.nih.gov/gene/3192090)

The TCS KdpD/KdpE, traditionally associated with potassium transport, has been demonstrated to play a role in the virulence and intracellular survival of many pathogenic bacteria (Freeman et al., 2013). KdpD/KdpE homologs have been identified in the *Francisella* genome; however, whether this system is responsible for potassium transport in *Francisella* has not been confirmed. Nevertheless, a mutant in either KdpD or KdpE in *F. novicida* is attenuated in animal models (Weiss et al., 2007; Moule et al., 2010; Freeman et al., 2013; Cuthbert et al., 2017). This phenotype may be unrelated to its role in potassium signaling, and has yet to be studied in human virulent *F. tularensis* serovars. Mutants in the SK KdpD gene were found to be attenuated following *F. tularensis* infection in the mouse model (Weiss et al., 2007); and KdpDE, as well as KdpAC, have been implicated in *F. novicida* infection in Drosophila (Moule et al., 2010).

In *Francisella*, no studies have been performed on the specific role of KdpDE in the regulation of potassium transport, osmolarity, or stress response. *Francisella* genomes also contain homologs of most of the components of the high-affinity potassium transporter pump KdpFABC (Table 3), except for the pseudogene of *kdpA* in SchuS4 (Alkhuder et al., 2010). The gene encoding the small protein KdpF found in the other genera is missing in the *Francisella* genomes, thus the role of this gene and the role of the activity of this potassium transporter remain to be studied in this organism. The *Francisella* genome also encodes low-affinity potassium transport proteins (the Trk system), which may still allow for potassium regulation in strains that lack the Kdp system (Alkhuder et al., 2010; Ireland et al., 2019).

**Figure 4:**
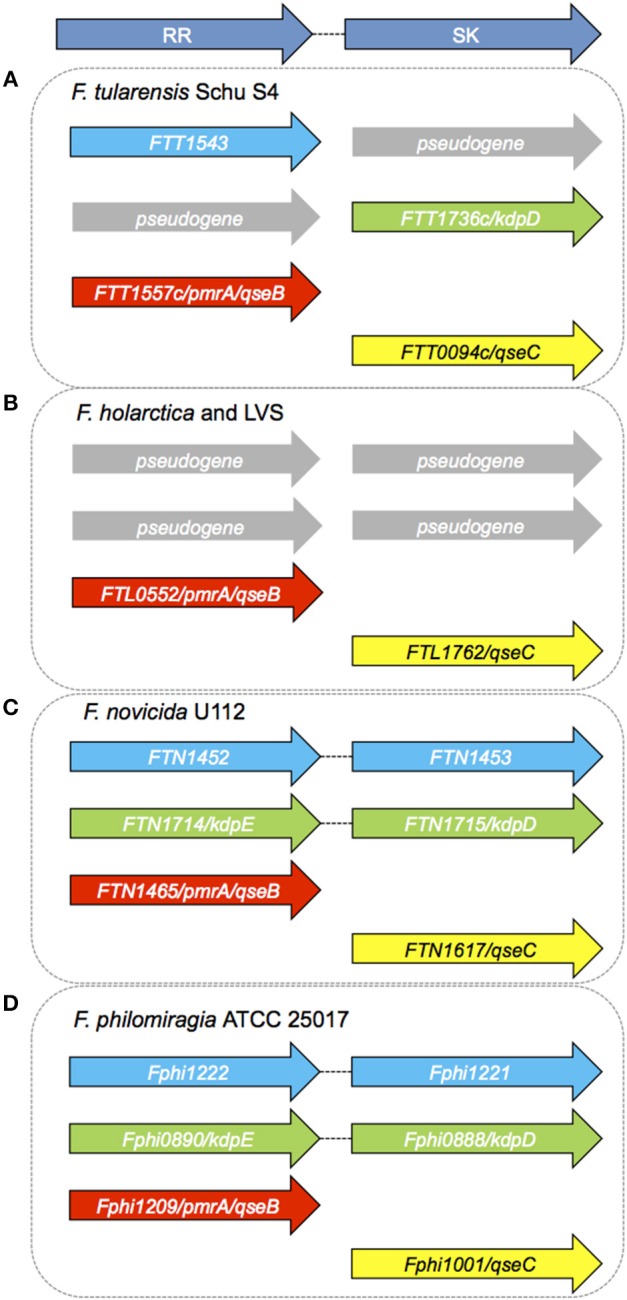
Sensor Kinases and Response Regulators in *Francisella* species. **(A)** There are no tandemly arranged TCS in *Ft* Schu S4 (Larsson et al., 2005). Both the SK *FTT1544* and RR *FTT1735c* (*kdpE*) are pseudogenes. *FTT1543, KdpD, PmrA/QseB*, and *QseC* are orphan TCS components in *F. tularensis* Schu S4. **(B)** There are no tandemly arranged TCSs in *F. holarctica* LVS. LVS (Live Vaccine Strain) has only one RR (*FTL0552/PmrA/QseB*) and one orphan SK (*QseC*). *F. holarctica* FSC200 has a similar set of genes as LVS, and has the same pseudogenes (Alkhuder et al., 2010). **(C)** There are two complete and one uncoupled TCS in *F. novicida. FTN1452/FTN1453* and *KdpDE* form the two tandemly arranged TCS, while *FTN1465* (*PmrA/QseB*) and *QseC* are orphan members. **(D)** *F. philomiragia* has three RR and three SK genes, similar to *F. novicida*. As in *F. novicida* and LVS, *PmrA/QseB*, and *QseC* appear to be orphan TCS components.

Environmental strains of *Francisella*, such as *F. novicida* and *F. philomiragia*, have an intact Kdp system, suggesting that this pathway could be active in these species since the SK-RR as well as the essential three components KdpABC of the high-affinity K+ transporter are present (Figure 4).

## External and Internal Small Molecule Signaling in *Francisella*

The survival of microbes in various environments is dependent on their intrinsic ability to detect and efficiently respond to changing extracellular signals. One such intrinsic sensory system in bacteria are the TCSs (Groisman, 2016). One of the best studied examples of a TCS in Gram-negative bacteria is the highly conserved is QseC/QseB system discussed above that is reported to detect proposed autoinducer-3 molecules (Clarke et al., 2006). Both QseC and QseB/PmrA homologs are present in the *Francisella* genome, but are not located together and co-transcribed like most TCSs. Thus, it has been proposed that QseC and QseB/PmrA are a non-classical, non-tandemly arranged TCS, in which each component are “orphans.” Environmental signals that activate this system in *Francisella* are unknown but are the subject of current investigation.

Bacteria also sense intracellular environmental conditions including small molecules and metabolites. In response to nutrient starvation and stress, some bacterial species produce hyper-phosphorylated guanosine diphosphate and triphosphate analogs termed (p)ppGpp. These small molecules bind to the β′- and ω-subunits of RNA polymerase (RNAP) to increase affinity of to the DNA target (Bell et al., 2010; Dalebroux and Swanson, 2012; Hauryliuk et al., 2015). (p)ppGpp regulates many bacterial processes including protein synthesis, bacterial replication, and acid stress response, which contributes to bacterial virulence and persistence (Hauryliuk et al., 2015). Production of (p)ppGpp is dependent on two bifunctional enzymes, RelA and SpoT, that synthesize (p)ppGpp from ATP, GTP, or GDP, and degrade (p)ppGpp to pyrophosphate and either GTP or GDP (Hauryliuk et al., 2015). While the RelA-dependent response is most often associated with amino acid starvation, a SpoT-dependent response is linked with various stress signals including fatty acid, iron, and carbon source starvation (Hauryliuk et al., 2015). Both *relA* and *spoT* genes are present in the *Francisella* genome (Larsson et al., 2005; Rohmer et al., 2007; Murch et al., 2017). Mutants defective in (p)ppGpp increased biofilm formation, reduced stress resistance, and are attenuated in macrophages and in the mouse model (Dean et al., 2009; Murch et al., 2017). In some bacteria, (p)ppGpp signaling interacts with TCSs to regulate gene expression (Nishino et al., 2005; Lamarche et al., 2008; Rifat and Karakousis, 2014). Charity et al. (2009) demonstrated that (p)ppGpp promotes interaction between PigR and the MglA-SspA complex that regulates the expression of FPI genes. Bell et al. (2010) showed that MglA-SspA complex also interacts with QseB/PmrA to regulate FPI gene expression. Of note, there are conflicting data in the literature regarding to this complex interaction, likely due to the different *Francisella* subspecies and growth conditions used in these studies (Brotcke and Monack, 2008; Bell et al., 2010; Ramsey and Dove, 2016; Cuthbert et al., 2017).

## Cross-Talk Between TCS Proteins

Although it is thought that SKs of paired TCSs will typically or preferentially phosphorylate their cognate RR, it is known that “promiscuity” between SKs and RRs can occur, in which they can “cross-talk” between different SKs and RRs (Yoshida et al., 2015; Groisman, 2016). Especially for organisms with very limited TCS genes and several orphan TCS genes, such as is observed in *Francisella*, it seems likely that promiscuous cross-phosphorylation could occur (Murch et al., 2017).

As previously mentioned, it has been demonstrated that inactivation of the unpaired QseC and QseB/PmrA genes dramatically reduces *Francisella* virulence in macrophages and in a mouse model of tularemia (Mohapatra et al., 2007; Rasko et al., 2008; Bell et al., 2010; Mokrievich et al., 2010) (Table 2). It has not yet been demonstrated if these two genes act as a functional paired TCS, and whether QseC phosphorylates QseB to alter gene expression. The conservation of both of these genes in the genus *Francisella* suggests that they are both important for this organism. Although the quorum-sensing ligand for *Francisella* QseC is not identified, by analogy to the *E. coli* QseC there may be a ligand (AI-3 for *E. coli*) that can activate the Histidine kinase activity of QseC (Walters and Sperandio, 2006; Kendall et al., 2007). Also by analogy, it is expected that QseC will not be activated in the absence of the ligand. It is interesting to note that the “sensing domain” of *Francisella* QseC and *E. coli* QseC differ in both length and sequence and thus may respond to different signals.

Bell et al. demonstrated that the SK KdpD is primarily responsible for phosphorylation of QseB/PmrA using isolated membrane fractions and the purified RR (Bell et al., 2010). Interestingly, in these experiments, the deletion of *qseC* did not alter the phosphorylation of QseB/PmrA. It could be that the proposed ligand for *Francisella* QseC was not present, and QseC would therefore be inactive under these conditions, while there may have been conditions resulting in KdpD activation, enabling it to phosphorylate QseB. Such complex networks of regulation of activation and phosphorylation would allow for complex responses to various environmental conditions (Weinberg et al., 2007).

## Role of TCS Signaling in *Francisella* Pathogenesis: a Target for New Therapeutics?

Developing new antimicrobials is challenging because of how quickly bacteria develop mechanisms of resistance. If we could identify agents that inhibit bacterial virulence without interfering in their growth, resistance could be slowed (Hung et al., 2005; Cegelski et al., 2008). TCS systems are potential targets for antimicrobial drug design since they are critical for antibiotic resistance and for virulence regulation in many Gram-negative bacteria (Hung et al., 2005; Cegelski et al., 2008; Curtis et al., 2014). In addition, TCS inhibitors may exert less toxicity to the host since the phosphorylation of Histidine of TCS in bacteria differs from serine/tyrosine/threonine phosphorylation signaling system in mammalian cells.

Several groups have demonstrated that inhibition of QseC signaling in *Francisella* is a promising approach to control *Francisella* infection (Rasko et al., 2008; Curtis et al., 2014; Dean and van Hoek, 2015). By screening an FDA-approved drug library, Dean and van Hoek identified several compounds that inhibit biofilm formation in *Francisella* (Dean and van Hoek, 2015). Notably, they showed that the antidepressant maprotiline inhibits both biofilm formation and FPI gene expression. Maprotiline rescued *F. novicida* infected wax worm larvae and prolonged the survival of *F. novicida* infected mice (Dean and van Hoek, 2015). As mentioned previously, the Sperandio laboratory identified a small molecule, LED209 that inhibits QseC through modification of conserved lysine residues (corresponding to K269 in FTT_0094c) (Curtis et al., 2014). As a consequence, LED209 is capable of inhibiting QseC-mediated activation of virulence genes and mouse virulence for *Salmonella typhimurium* and *F. tularensis* SchuS4 (Rasko et al., 2008; Curtis et al., 2014). In addition, Wrench et al. identified that quinacrine affects the interaction between MglA and SspA, which altered the intramacrophage survival of *F. novicida* (Wrench et al., 2013).

Targeting the response regulator is also an area of active research. Milton et al. (2017) resolved the structure of the N-terminal receiving domain of QseB/PmrA and it has been experimentally demonstrated that binding of 2-aminoimidazole (2AI) based compounds inhibited *Francisella* biofilm formation and induced antibiotic re-sensitization in *Francisella* and other bacteria (Stephens et al., 2016; Milton et al., 2017, 2018).

Taken together, targeting *Francisella* TCSs or accessory transcription factors presents a potential therapeutic approach for controlling human tularemia.

## Remaining Questions Regarding TCS in *Francisella*

Some interesting questions remain to be addressed with regard to two-component systems in the genus *Francisella*.

➢ What are the activating signals for *Francisella* HKs? How does the signaling through the orphaned TCSs change in the presence of the activating ligand(s) for the SK?
➢ How is the signaling integrated between QseC, KdpD, and QseB/PmrA in the human virulent strains?
➢ What is the role of KdpDE in the regulation of potassium transport and osmotic stress in *Francisella*? How is potassium transport regulated in the human virulent strains that lack KdpE?
➢ Does the potassium transport system KdpABC play a role in pathogenesis in *Francisella*?
➢ For environmental strains, how is their signaling and gene regulation different from the human virulent serovars, with their fuller complement of intact TCSs?
➢ What role do sRNAs play in compensating for or augmenting the function of TCSs in *Francisella*?
➢ Can targeting of TCSs be an effective therapeutic for *F. tularensis* infections?

## Summary of Two-Component Systems in *Francisella*

Overall there are very few (and often not intact) classically paired TCSs in *Francisella* strains and species, and relatively few TCS genes overall in the genus (Table 1; Supplemental Table 1). All *Francisella* genomes examined to date contain QseB/PmrA and QseC (Figure 4; Supplemental Table 1), suggesting that these two highly conserved genes play essential roles in the life-cycle of this organism. QseB/PmrA and QseC are always orphan TCS members due to their unpaired locations in the genome. KdpD is an orphan RR in some strains, such as *F. tularensis* SchuS4, in which the cognate SK is a pseudogene and is absent in the Live Vaccine Strain. The dependence of all *Francisella* strains on orphan TCS components suggests that intra-molecular communication between the components of the different TCS will likely be a determining factor in their physiological roles. On the other hand, it may suggest that they function in a unique way in *Francisella*—either with promiscuity among the SK and RR proteins or that phosphotransfer occurs differently or perhaps is not integral to their function. In the absence of large numbers of other TCS proteins, the relative simplicity of the *Francisella* TCSs makes this bacterium an ideal model organism for investigating the role of these key regulatory factors.

The presence of intact TCSs in the environmental strains of *Francisella*, such as *F. novicida, F. philomiragia*, and *F. noatunensis*, is notable compared to the number of incomplete and disrupted TCSs in the human pathogenic strains (Figure 4). *F. philomiragia* has two intact classical TCS operons (KdpDE and the Unnamed RR/SK) plus QseB and QseC, as do *F. novicida* and *F. noatunensis*. While the precise role of the TCS genes in environmental persistence and survival compared to their role in virulence and pathogenesis is not clearly understood for *Francisella*, this clear difference highlights the need for further research into these signaling and regulatory systems. This area of research will yield important insights into the regulation and integration of environmental and host-induced stressors in the genus *Francisella*.

With a greater understanding of its TCSs and the application of current technologies in gene expression, proteomics and post-translational modifications, we can advance our understanding of the regulation of gene expression in environmental and pathogenic strains of *Francisella*. In addition, new opportunities may be identified to develop structurally designed therapeutic interventions against *Francisella*'s TCS proteins.

## Author Contributions

MH and JG conceived the idea for the review and co-wrote the manuscript with KH.

### Conflict of Interest Statement

The authors declare that the research was conducted in the absence of any commercial or financial relationships that could be construed as a potential conflict of interest.
