# Supplementary material for: Two-Component Systems in Francisella Species
Source: Front Cell Infect Microbiol. 2019 Jun 12;9:198. doi: 10.3389/fcimb.2019.00198 (PMC6584805; doi:10.3389/fcimb.2019.00198)
Supplement: Supplementary file 1 [file Table_1.docx]

**Supplemental Table 1: List of Two Component System gene homologs in many species of the genus *Francisella.*** Data for this table was assembled from UniProt ([www.uniprot.com](http://www.uniprot.com)). Locus numbers are indicated first and in bold, UniProt accession numbers are listed second. Genes (and proteins) are homologous by sequence comparison to *F. novicida* U112 genes (between 50-100% identical).

| **Species** | **QseC** | **QseB** | **KdpD** | **KdpE** | **Unnamed SK** | **Unnamed RR** | **Other** |
| --- | --- | --- | --- | --- | --- | --- | --- |
| *F. tularensis SchuS4* | **FTT_0094c, BZ14_794**  [Q5NIH6](https://www.uniprot.org/uniprot/Q5NIH6) | **FTT_1557c, BZ14_1198**  [Q5NER1](https://www.uniprot.org/uniprot/Q5NER1) | **FTT_1736c, BZ14_978**  [Q5NEA7](https://www.uniprot.org/uniprot/Q5NEA7) | **Pseudogene** | **Pseudogene** | **FTT1543**  BZ14_1215  [Q5NES1](https://www.uniprot.org/uniprot/Q5NES1) |  |
| *F. tularensis LVS* | **FTL_1762**  **DA46_965**  [A0A0B6CNG4](https://www.uniprot.org/uniprot/A0A0B6CNG4) | **FTL_0552**  **DA46_133,**  [A0A0B6E4M4](https://www.uniprot.org/uniprot/A0A0B6E4M4) | **Pseudogene** | **Pseudogene** | **Pseudogene** | **Pseudogene** |  |
| [*Francisella tularensis subsp. novicida (strain U112)*](https://www.uniprot.org/taxonomy/401614) | **FTN_1617**  [A0Q8B1](https://www.uniprot.org/uniprot/A0Q8B1) | **FTN_1465**  [A0Q7W8](https://www.uniprot.org/uniprot/A0Q7W8) | **FTN_1715**  [A0Q8K7](https://www.uniprot.org/uniprot/A0Q8K7) | **FTN_1714**  [A0Q8K6](https://www.uniprot.org/uniprot/A0Q8K6) | **FTN_1453**  A0Q7V6 | **FTN_1452**  A0Q7V5 |  |
| [*Francisella tularensis subsp. novicida PA10-7858*](https://www.uniprot.org/taxonomy/1386968) | **N894_1808**  A0A1I9YBV9 | **N894_1658**  [A0A1I9YBF9](https://www.uniprot.org/uniprot/A0A1I9YBF9) | **N894_1908**  [A0A1I9YC59](https://www.uniprot.org/uniprot/A0A1I9YC59) | **N894_1907**  [A0A1I9YC58](https://www.uniprot.org/uniprot/A0A1I9YC58) | **N894_1646**  A0A1I9YBE7 | **N894_1645**  A0A1I9YBE6 |  |
| *F. philomiragia subsp. philomiragia 25017* | **Fphi_1001**  [B0TWW8](https://www.uniprot.org/uniprot/B0TWW8) | **Fphi_1209**  B0TXH6 | **Fphi_0888**  [B0TWK3](https://www.uniprot.org/uniprot/B0TWK3) | **Fphi_0890**  [B0TWK4](https://www.uniprot.org/uniprot/B0TWK4) | **Fphi_1221**  B0TXI8 | **Fphi_1222**  [B0TXI9](https://www.uniprot.org/uniprot/B0TXI9) | **RR** NtrX  **Fphi_0797**  [B0TWB1](https://www.uniprot.org/uniprot/B0TWB1)  **SK**  **Fphi_0794**  [B0TWA8](https://www.uniprot.org/uniprot/B0TWA8) |
| [*Francisella philomiragia subsp. philomiragia ATCC 25015*](https://www.uniprot.org/taxonomy/539329) | **BZ13_1037**  [C6YU85](https://www.uniprot.org/uniprot/C6YU85) | **BZ13_796**  C6YUL5 | **BZ13_1144**  [C6YTP5](https://www.uniprot.org/uniprot/C6YTP5) | **BZ13_1142**  [C6YTP6](https://www.uniprot.org/uniprot/C6YTP6) | **BZ13_783**  A0A0B6ENN4 | **BZ13_782**  C6YUM8 |  |
| [*Francisella philomiragia*](https://www.uniprot.org/taxonomy/28110) | **LA55_1888**  [A0A0B6D3F6](https://www.uniprot.org/uniprot/A0A0B6D3F6) | **LA55_1688**  [A0A0B6CS10](https://www.uniprot.org/uniprot/A0A0B6CS10) | **LA55_2011**  [A0A0B6D2K9](https://www.uniprot.org/uniprot/A0A0B6D2K9) | **LA55_2009**  [A0A0B6D3Y5](https://www.uniprot.org/uniprot/A0A0B6D3Y5) | **LA55_1689**  A0A0B6D1F0 | **LA55_1688**  A0A0B6CS10 |  |
| *Francisella noatunensis subsp. noatunensis FSC772* | **FSC772_08490**  [A0A248KQI8](https://www.uniprot.org/uniprot/A0A248KQI8) | **FSC772_07605**  [A0A248KR40](https://www.uniprot.org/uniprot/A0A248KR40) | **FSC772_09040**  A0A248KQU7 | **FSC772_09030**  A0A248KSS7 | **FSC772_07545**  A0A248KQX7 | **FSC772_07540**  A0A248KRM8 |  |
| [*Francisella sp. (strain TX077308)*](https://www.uniprot.org/taxonomy/573569) | **F7308_0605**  [F8GAJ7](https://www.uniprot.org/uniprot/F8GAJ7) | **F7308_0805**  [F8GBG5](https://www.uniprot.org/uniprot/F8GBG5) | **F7308_0486**  [F8G9R9](https://www.uniprot.org/uniprot/F8G9R9) | **F7308_0487**  [F8G9S0](https://www.uniprot.org/uniprot/F8G9S0) | **F7308_0817**  F8GBH7 | **F7308_0818**  F8GBH8 |  |
| [*Francisella hispaniensis*](https://www.uniprot.org/taxonomy/622488) | **FN3523_1678**  [F4BHN7](https://www.uniprot.org/uniprot/F4BHN7) | **FN3523_1532**  [F4BH91](https://www.uniprot.org/uniprot/F4BH91) | **FN3523_1787**  F4BHZ6 | **FN3523_1786**  [F4BHZ5](https://www.uniprot.org/uniprot/F4BHZ5) | **FN3523_1520** F4BH79 | **FN3523_1519**  F4BH78 |  |
